# BIRC5 is a prognostic biomarker associated with tumor immune cell infiltration

**DOI:** 10.1038/s41598-020-79736-7

**Published:** 2021-01-11

**Authors:** Linlong Xu, Wenpeng Yu, Han Xiao, Kang Lin

**Affiliations:** 1 Department of Hepato-Biliary-Pancreatic Surgery, Jiujiang First People’s Hospital, Jiujiang, 332000 China; 2 Department of Cardiothoracic Surgery, The Second Affiliated Hospital of Nanchang University, Nanchang, 330006 China; 3 Hepato-Biliary-Pancreatic Surgery Division, Department of General Surgery, The Second Affiliated Hospital of Nanchang University, No.1, Minde Road, Nanchang, 330006 China; 4 Gastrointestinal Surgery Division, Department of General Surgery, The Second Affiliated Hospital of Nanchang University, No.1, Minde Road, Nanchang, 330006 China

**Keywords:** Prognostic markers, Cancer

## Abstract

BIRC5 is an immune-related gene that inhibits apoptosis and promotes cell proliferation. It is highly expressed in most tumors and leads to poor prognosis in cancer patients. This study aimed to analyze the relationship between the expression level of BIRC5 in different tumors and patient prognosis, clinical parameters, and its role in tumor immunity. Genes co-expressed with BIRC5 were analyzed, and functional enrichment analysis was performed. The relationship between BIRC5 expression and the immune and stromal scores of tumors in pan-cancer patients and the infiltration level of 22 tumor-infiltrating lymphocytes (TILs) was analyzed. The correlation of BIRC5 with immune checkpoints was conducted. Functional enrichment analysis showed that genes co-expressed with BIRC5 were significantly associated with the mitotic cell cycle, APC/C-mediated degradation of cell cycle proteins, mitotic metaphase, and anaphase pathways. Besides, the high expression of BIRC5 was significantly correlated with the expression levels of various DNA methyltransferases, indicating that BIRC5 regulates DNA methylation. We also found that BIRC5 was significantly correlated with multiple immune cells infiltrates in a variety of tumors. This study lays the foundation for future research on how BIRC5 modulates tumor immune cells, which may lead to the development of more effective targeted tumor immunotherapies.

## Introduction

Cancer poses a severe threat to human health and has a high mortality rate. The three most common cancers among men are prostate, colon and rectum, and skin melanoma. Among women, breast, uterine body, and colon and rectum are the most prevalent cancers^1^. Early detection and effective treatment will improve the survival rates of cancer patients. Currently, the most common treatments for cancer are surgical resection, radiation therapy, and adjuvant chemotherapy, but their efficacy is still limited^2^. Immunotherapy has recently emerged as an effective cancer treatment option. Some of the newer approaches of immunotherapy include immune checkpoint blockade and chimeric antigen receptor T (CAR T) cell therapy. These approaches have attracted much attention in cancer immunotherapy and are thought to cure various cancers^3,4^. BIRC5, also known as survivin, is an immune-related gene and member of the apoptotic (IAP) protein family. Overexpression of BIRC5 in cancer may inhibit this apoptotic checkpoint and favor aberrant mitosis of transformed cells^5^. In recent years, several studies have reported the role of BIRC5 in cancer^6,7^, but the role of BIRC5 in pan-cancer or its impact on the immune microenvironment has not been investigated.

Bioinformatics techniques were used in this study to predict BIRC5 affects the prognosis of pan-cancer and infiltration of immune cells in tumors. The relationship between BIRC5 and known immune checkpoints as well as the correlation of BIRC5 expression levels with the expression of four methyltransferases (DNMT1, DNMT2, DNMT3A, DNMT3B) in different tumors was also analyzed. GSEA enrichment analysis was conducted to reveal how BIRC5 regulates different tumors. The results provided strong evidence that BIRC5 might be an antitumor agent. Thus, BIRC5 can be considered an immune prognostic marker with potential application in immunotherapies.

## Result

### Transcriptional levels of BIRC5 in pan-cancer

**Figure 1:**
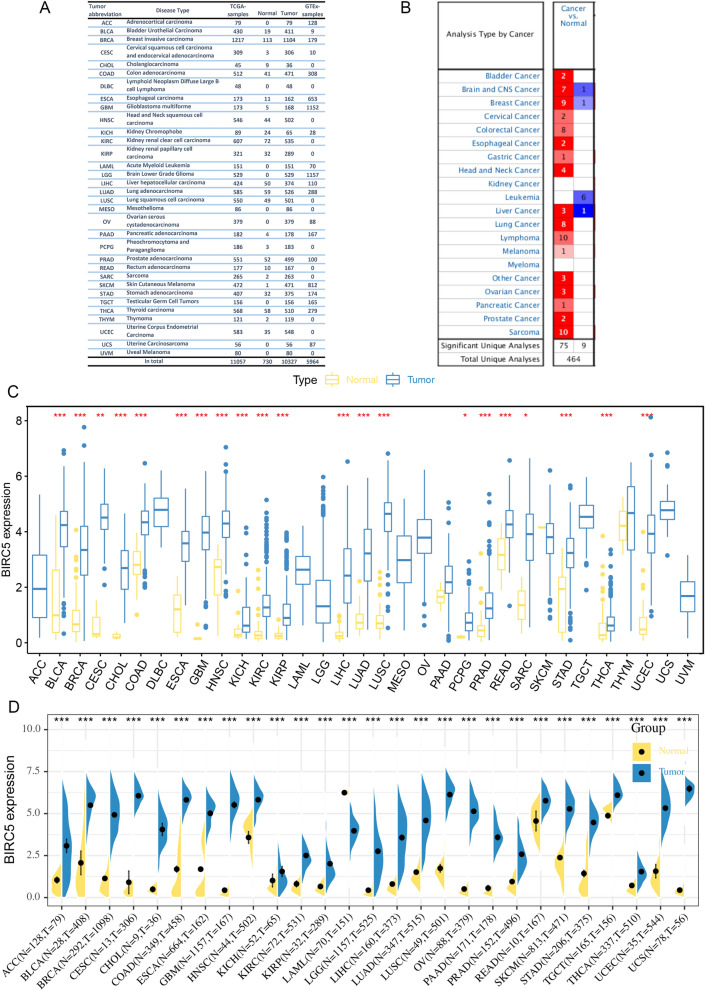
The expression level of BIRC5 in cancer patients. (**A**) The number of normal tissue samples and cancer tissue samples of 33 kinds of cancer patients in TCGA database, and the number of normal tissue samples of 33 kinds of tumors in GTEx database. (**B**) The difference of BIRC5 expression between cancer tissues and normal tissues of patients with 20 kinds of cancers in Oncomie database. (**C**) The difference of BIRC5 expression between cancer tissue and normal tissue in 33 kinds of cancer patients in TCGA database. (**D**) The difference of BIRC5 expression between cancer tissue and normal tissue of 33 kinds of cancer patients was analyzed by TCGA and GTEx database. *P < 0.05. *P < 0.01. *P < 0.001. The figure was performed using R version 3.6.1 (2019-07-05)^46^.

We collected transcriptome data from 33 tumor patients in the TCGA and GTEx databases, including 11,057 cases in the TCGA database and 5964 cases in the GTEx database (Fig. 1A). In the Oncomine database, we found that BIRC5 was highly expressed in 18 of the 20 tumor tissues included (Fig. 1B). Analysis of expression profiling data of tumor tissues and healthy tissues of patients with different cancers in the TCGA database revealed that BIRC5 was highly expressed in 21 out of 33 tumor tissues (Fig. 1C). The transcription levels of BIRC5 in different cancers were determined by joint analysis of pan-cancer data in TCGA matched with normal samples in GTEx. Except for Leukemia, BIRC5 mRNA was highly expressed in all tumor tissues (Fig. 1D).

### Prognostic value of BIRC5 in pan-cancer

**Figure 2:**
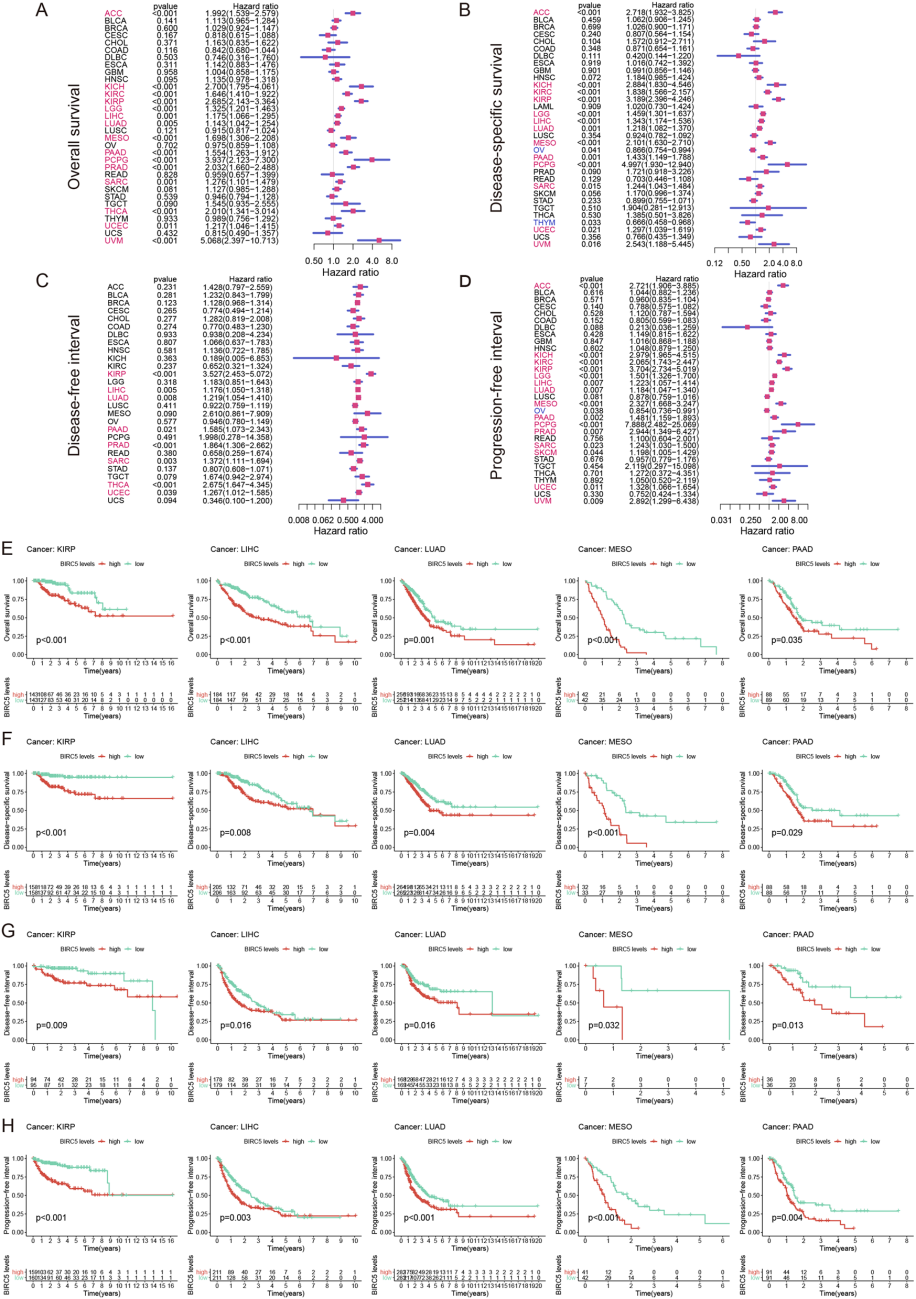
The relationship between the expression of BIRC5 and the prognosis of patients with 33 kinds of cancers in TCGA database. (**A**) Overall survival. (**B**) Disease-specific survival. (**C**) Disease-free interval. (**D**) Progression-free interval. Five kinds of tumors whose prognosis is most related to the difference of BIRC5 expression including (**E**) Overall survival, (**F**) Disease-specific survival, (**G**) Disease-free interval, (**H**) Progression-free interval**.** The figure was performed using R version 3.6.1 (2019-07-05)^46^.

The prognosis of each tumor sample in the TCGA database was analyzed to determine the effect of BIRC5 expression levels on the prognosis of patients with different tumors. The results showed that high expression of BIRC5 was positively correlated with poor overall survival in 15 tumors (Fig. 2A), poor disease-specific survival in 13 tumors (Fig. 2B) and poorer disease-free interval in patients with 8 tumors (Fig. 2C). It was positively correlated with poorer progression-free interval in 15 tumors (Fig. 2D). Taken together, these results showed that high expression of BIRC5 correlated with poor prognosis in patients with KIRP, LIHC, LUAD, MESO, and PAAD (Fig. 2E-H).

### The relationship of BIRC5 with clinicopathological features

**Figure 3:**
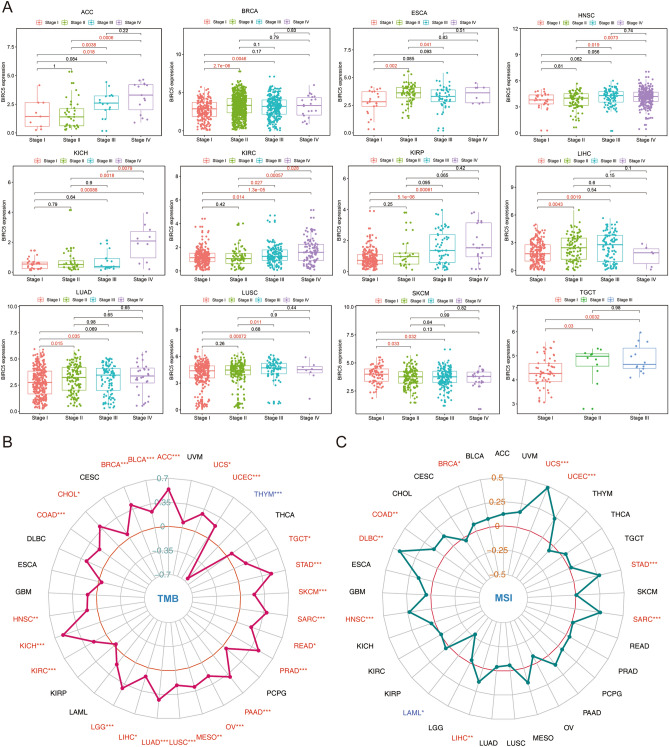
The relationship between BIRC5 expression level and pathological characteristics of tumor patients the relationship between. (**A**) BIRC5 expression level and Stage grade of cancer patients the relationship between. (**B**) BIRC5 expression level and Tumor mutation burden (TMB) in cancer patients the relationship between. (**C**) BIRC5 expression level and microsatellite instability (MSI) in cancer patients. *P < 0.05. *P < 0.01. *P < 0.001. The figure was performed using R version 3.6.1 (2019-07-05)^46^.

The relationship between BIRC5 expression levels and the clinicopathological characteristics of patients with different tumors was analyzed based on stage pathology grade, tumor mutation burden (TMB), and microsatellite instability (MSI) status of tumor samples from the TCGA database. Results showed that in the vast majority of tumors, the higher the stage grade, the higher the expression level of BIRC5 in patients (Fig. 3A). Moreover, the expression level of BIRC5 in 24 tumors such as ACC, UCEC, STAD, SKCM, and SARC was significantly correlated with TMB (Fig. 3B), whereas it was significantly correlated with MSI in 10 tumors such as UCS, UCEC, STAD, and SARC (Fig. 3C). Therefore, BIRC5 may be a prognostic marker for multiple tumors.

### The relationship between BIRC5 and the tumor microenvironment

The estimate package was used to analyze the immune score and stromal score of each tumor sample and determine the relationship between BIRC5 expression level, immune score, and stromal score in 33 tumors. Results showed that the immune scores of KIRC, THCA, and THYM were positively and negatively correlated with BIRC5 expression levels, while those of ESCA, GBM, LUSC, STAD, and UCEC were negatively and positively correlated with BIRC5 expression levels (Fig. S1A). By contrast, the stromal scores of BIRC, COAD, GBM, HNSC, LIHC, LUAD, LUSC, SARC, SKCM, STAD, THY, and UCEC were significantly negatively correlated with the expression level of BIRC5 (Fig. S1B).

### The association of BIRC5 with tumor immune cell infiltration levels

**Figure 4:**
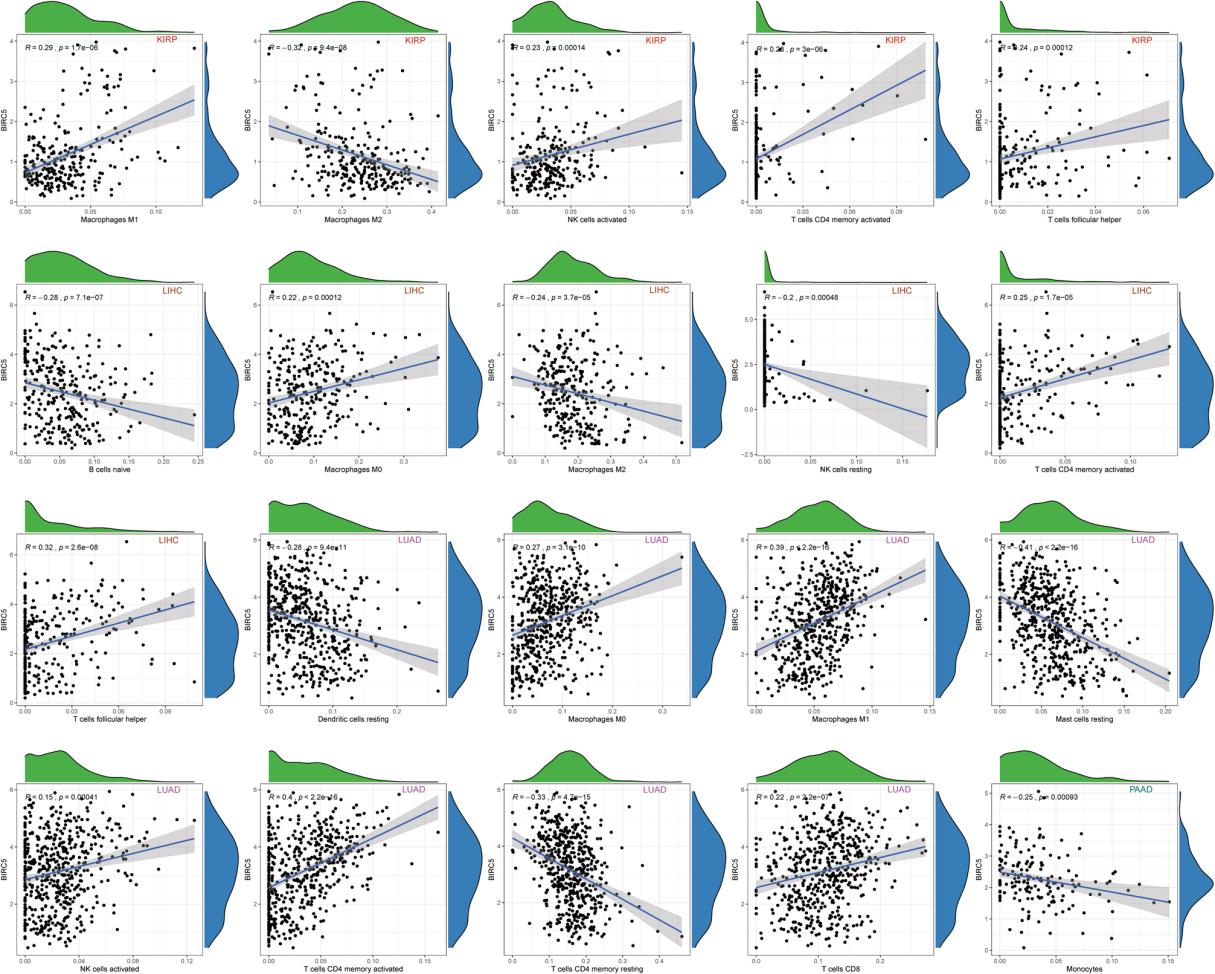
The relationship between the expression level of BIRC5 and the infiltration level of different kinds of immune cells in cancer patients. The figure was performed using R version 3.6.1 (2019-07-05)^46^.

Given that immune infiltrating cells play a significant role in cancer development, we investigated the relationship between BIRC5 expression levels and immune cell infiltration in different types of cancers. Data on the scores of 22 immune infiltrating cells from 33 cancers were downloaded from the TIMER (https://cistrome.shinyapps.io/timer/) database. Using these data, the correlation between BIRC5 expression levels and infiltration levels of these immune cells was analyzed separately. High BIRC5 expression in KIPP was positively correlated with the infiltration levels of macrophages M1, NK cells activated, T cells CD4 memory activated, T cells follicular helper, and macrophages M2, NK cells activated, T cells CD4 memory activated, and T cells follicular helper. High BIRC5 expression in KIPP was negatively correlated with the infiltration level of B cells naïve, macrophages M0, T cells follicular helper, and NK cells. High expression of BIRC5 in LIHC was positively correlated with the infiltration level of macrophages M0, T cells follicular helper, and negatively correlated with the infiltration level of B cells naïve, macrophages M2, NK cells resting. High expression of BIRC5 in LUAD was positively correlated with infiltration levels of macrophages M0, macrophages M1, NK cells activated, T cells CD4 memory activated, T cells CD8, and dendritic cells resting, mast cells resting, and T cells CD4 memory resting were negatively correlated. High expression of BIRC5 in PAAD was negatively correlated with the infiltration level of monocytes (Fig. 4). Furthermore, we found that high expression of BIRC5 in different types of tumors was positively correlated with the activation of multiple immune cells and negatively correlated with the resting state of immune cells (Fig. S2).

### Functional enrichment analysis of BIRC5

**Figure 5:**
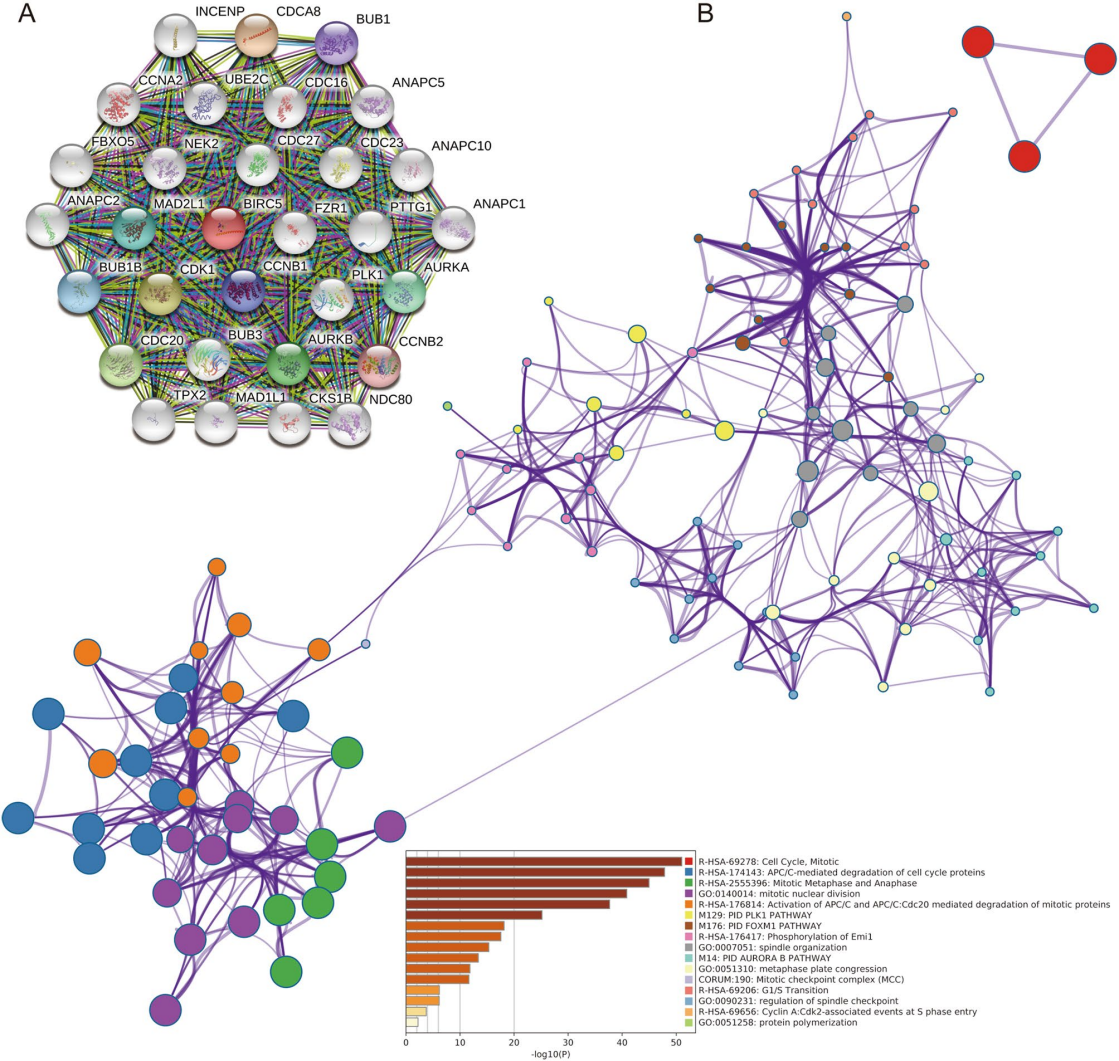
Potential molecular mechanisms and functional enrichment analysis of BIRC5. (**A**) The PPI network of BIRC5-related genes. (**B**) Functional enrichment Analysis based on BIRC5 interacting proteins. The figure was performed using R version 3.6.1 (2019-07-05)^46^.

**Figure 6:**
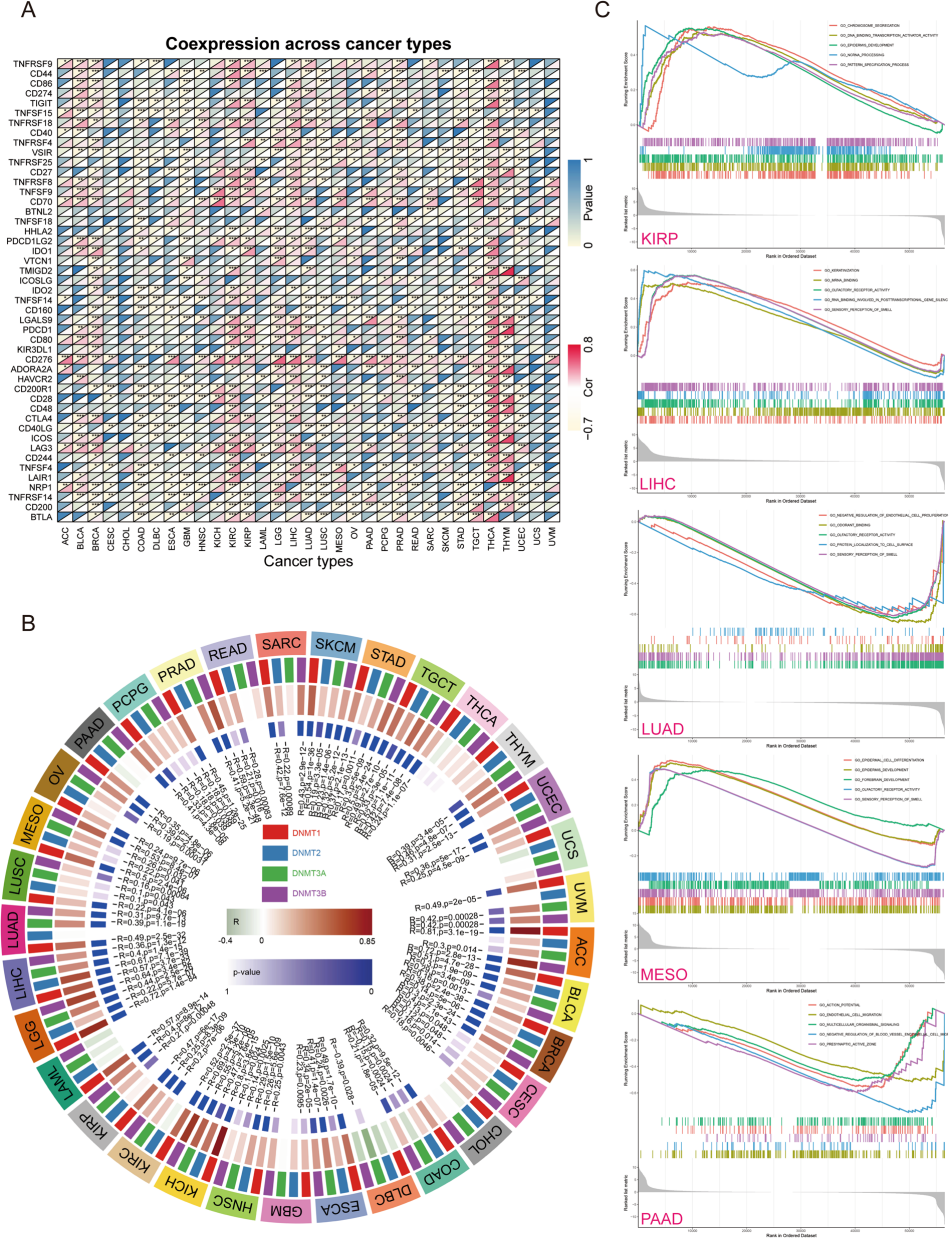
The relationship between BIRC expression and immune related genes. (**A**) The correlation between BIRC5 expression level and immune checkpoint gene expression. (**B**) The relationship between the expression level of BIRC5 and 4 methyltransferases. (**C**) GSEA analysis of BIRC5 in five kinds of tumors whose prognosis is closely related to the expression of BIRC5. The figure was performed using R version 3.6.1 (2019-07-05)^46^.

To investigate the molecular mechanisms through which BIRC5 regulates various cancers, we constructed a protein–protein interaction (PPI) network (Fig. 5A) of 30 BIRC5-associated proteins using the STRING database. Functional enrichment analysis showed that the 30 BIRC5-associated proteins were mainly enriched in the cell cycle, mitotic, APC/C-mediated degradation of cell cycle proteins, mitotic metaphase, and anaphase (Fig. 5B). Tumor immunotherapy controls and clears tumors by reactivating and maintaining the tumor-immune cycle and restoring the body's normal antitumor immune response. Here we analyze the relationship between BIRC5 expression levels and the expression of immune checkpoint genes using more than forty common immune checkpoint genes. The results showed that BIRC5 expression levels correlated with several immune checkpoint genes in different types of tumors (Fig. 6A). DNA methylation is a form of chemical modification of DNA that can affects gene expression without altering the DNA sequence. It can also cause changes in chromatin structure, DNA conformation, DNA stability, and the manner in which DNA interacts with proteins, thereby regulating gene expression. Here we analyzed the correlation between the expression level of BIRC5 and the expression of four methyltransferases (DNMT1, DNMT2, DNMT3A, and DNMT3B) in different tumors. The results showed that the high expression of BIRC5 was correlated with the expression of methyltransferase in tumors except UCS and READ. (Fig. 6B). In further analysis, we divided tumor samples into two groups based on the median expression of BIRC5. GSEA analysis showed that high expression of BIRC5 promoted chromosome activity, DNA binding specification transcription process, epidermal development, ncRNA transcription pattern in KIRP. It promoted keratinization, mRNA binding, olfactory receptor activity, RNA binding in posttranscriptional gene silencing, and sensory perception of smell in LIHC. It promotes epidermal cell differentiation, epidermal development, forebrain development, olfactory receptor activity, and sensory perception activity in MESO. However, high expression of BIRC5 inhibits the proliferation of endothelial cells, odorant binding, olfactory receptor activity, protein localization of cell surface, sensory perception by cell surfaces, and perception of smell in LUAD. It inhibits the action potential, endothelial cell migration, multicellular organismal signaling, negative regulation of blood endothelial vessels, active presynaptic zone in PAAD (Fig. 6C). BIRC5 affects the development in different tumors through diverse pathways (Fig. S3), and thus it has the potential to serve as a molecular marker in different tumors.

## Discussion

In this study, BIRC5 was highly expressed in all solid tumor tissues. These findings are consistent with those reported in a previous study^8^. Furthermore, BIRC5 expression was relatively high in advanced tumor stages in KIRC, KIRP, LIHC, LUAD and UCEC, and relatively low in advanced tumor stages in STAD. In KIRC, STAD, and UCEC, BIRC5 expression was higher in advanced tumor stages. Several studies have confirmed the prognostic role of BIRC5 in a variety of tumors. Su et al. investigated the role of BIRC5 in hepatocellular carcinoma and reported that octamer-binding transcription factor 44 (OCT4) enhanced the expression of BIRC5 via cyclin D1 (CCND1). This promoted the proliferation of hepatocellular carcinoma cells and reduced their susceptibility to chemoradiotherapy, leading to poor prognosis^9^. BIRC5 is highly expressed in lung cancer and enhances the prognostic value of platinum-based therapies by decreasing BIRC5 expression^10^. High expression of BIRC5 is associated with poor prognosis in patients with hepatocellular and pancreatic cancer, lung cancer, renal papillary cell carcinoma, renal clear cell carcinoma, endometrial carcinoma, and sarcoma. However, patients with gastric and ovarian serous cystic carcinoma have been reported to have a better prognosis. Therefore, BIRC5 is an important prognostic biomarker for these tumors.

A study by T A Chan et al. found that TMB can be used as an immunotherapy biomarker and that high TMB can benefit immune checkpoint blockade (ICB) therapy^11^. Our study found that high expression of BIRC5 was significantly associated with TMB and high MSI in a variety of tumors, and correlation studies have also shown that BIRC5 expression levels correlate with the mutational load of breast cancer^12^. High Survivin expression in the presence of high MSI is an indicator of poor prognosis of FIGO stage I endometrial-like adenocarcinoma^13^. High expression of BIRC5 may contribute to breast tumor proliferation by promoting genetic instability^14^. These are consistent with our study so that in other tumors, BIRC5 is associated with the relationship between TMB and high MSI that deserves further study. The association of BIRC5 with TMB and MSI found in our study is novel in various tumors. BIRC5 has potential as a therapeutic target for ICB in cancer patients, and the relationship between BIRC5 and high TMB and high MSI warrants more in-depth study.

DNA methylation is a form of chemical modification of DNA, which changes the genetic expression without altering the DNA sequence. DNA methylation cause changes in chromatin structure, DNA conformation, DNA stability, and DNA–protein interaction, and hence regulate gene expression and tumor development. It has been shown that the expression levels of DNA methyltransferase 1 (DNMT1), Dnmt3b and Dnmt1/Dnmt3a can regulate the methylation status of BIRC5^15^. DNMT1 regulates BIRC5^16^, and overexpression of DNMT 1 can induce DNA methylation after BIRC5 silencing^17^. However, the relationship between the expression levels of DNMT2, DNMT3A, DNMT3B, and BIRC5 has not been investigated. In our study, we found that the correlation between expression levels of DNA methyltransferase and BIRC5 varied in different tumors. Hence, further studies are needed to explore how BIRC5 regulate DNA methyltransferase.

In recent years, studies have shown that BIRC5 may be a universal target antigen for anti-cancer immunotherapy. In our study, we found that the expression level of BIRC5 was positively correlated with the activation status of NK cells and CD4 T cells, but negatively correlated with the dormancy status of naive B cells, dendritic cells, CD4 T cells, and mast cells. Furthermore, results showed that down-regulation of BIRC5 severely affected tumor cells’ viability, indicating that it can be an important candidate for anti-cancer therapeutic vaccines^18^. We also found that it elicits CD8(+) T-cell-mediated responses in peripheral blood or tumor-associated lymphocytes from patients at different disease stages. BIRC5-specific T lymphocytes which recognize colorectal cancer cells and BIRC5-specific class I HLA-restricted T lymphocytes were activated and released interleukin 2 in response to HLA/ BIRC5-peptide complexes expressed by tumor cells. In addition to the CD8-mediated response, survivin specifically stimulated CD4+ in peripheral blood lymphocytes from the same patients' T cell reactivity^19^. Asanuma et al. demonstrated that BIRC5 up-regulates FasL expression and enables cancer cells to suppress Fas-mediated apoptotic signals and attack immune cells by inducing FasL^20^. Macrophages play an essential role in tumors, and different subtypes of macrophages have different markers. CD68 and CD163 are surface markers of macrophage-M0^21^. CD86 is an ideal marker of Macrophage-M1^22^, while CD206 is a landmark antigen of Macrophage-M2^23^. Our finding that BIRC5 expression in KIRP, LIHC, LUAD, and PAAD was significantly correlated with infiltration of multiple immune cells indicate that BIRC5 may be a potential immunotherapeutic target in these tumors.

Because BIRC5 is highly expressed in a variety of tumors, promote tumor progression, and influence immune cell status, researchers have used different means to target BIRC5 in cancer patients. Notably, it has been found that BIRC5-induced specific T-cell reactivity is strongly correlated with tumor response and patient survival, suggesting that vaccination with BIRC5-derived peptides is a promising therapeutic strategy for melanoma^24^. A vaccine designed against BIRC5 HLA class I peptide generated strong antigen-specific immune responses in ovarian cancer patients^25^. CTL activity and tumor growth inhibition was significantly enhanced in vivo in mice vaccinated with a combination of MUC1 and BIRC5 tumor gene vaccine. The CTL activity response was enhanced by nearly 200% and further enhanced by nearly 60% when combined with IL-2 adjuvant^26^. Liu et al. constructed a combination gene tumor vaccine from MUC1 and survivin (MS). The sPD1 / MS fusion DNA vaccine increased the specific cytolysis rate from 21.64 to 34.77%. In a mouse model of colorectal cancer, the sPD1/MS vaccine increased tumor suppression rate from 17.18 to 30.96% and prolonged the survival from 6.96 to 19.44%. The combination of sPD1/MS vaccine and oxaliplatin increased tumor suppression to 74.71% in a mouse model of colorectal cancer. The sPD1/MS vaccine exhibited promising anti-tumor effects, increased levels of tumor-infiltrating CD8T cells by 6.5-fold (from 0.10 to 0.65%) in a mouse model of lung cancer. It also increased the level of tumor-infiltrating CD8T cells by 6.5-fold (from 0.10 to 0.65%) in a mouse model of colorectal cancer by potently activating the tumor-suppressing immune system. Lymphocytes show good immunogenicity and anti-tumor effects^27^. Targeting heterologous BIRC5 to mature dendritic cells in lymphoid tissues induced strong human and mouse survivin-specific CD4 T-cell responses^28^. Multiepitope cancer vaccines prepared against BIRC5 in recent years inhibit tumor growth and strongly suppress lung metastasis. We demonstrated that the vaccine-induced broad cellular immune responses, accompanied by T cell infiltration^29^. Several studies have shown that vaccines targeting BIRC5 have promising anti-tumor effects through different approaches.

The PPI network shows that BIRC5 energetically interacts with 30 genes (including CCNB1, CDCA8, FBXO5, PLK1, and UBE2C). This specific intrinsic link between BIRC5 and interacting genes may be crucial to their role in tumor progression. It may be possible that multiple genes interact to regulate tumor progression. Further functional enrichment analysis showed that BIRC5 and interacting genes are mainly involved in the cell cycle, mitosis, APC/C-mediated degradation of cyclins, PLK1 signaling pathway, and FOXM1 signaling pathway. In mammals, mitosis and apoptosis maintain a relative balance in the total number of cells normal tissue function. However, disturbing the balance leads to the possibility of tumor development^30^. It has been found that expression of both BIRC5 and Plk1 is out of control in cancer, and disruptions to survivin or PLK1 activity show many similarities, thus linking them in cell division and cell death^31^. Further studies have shown that PLK1 promotes phosphorylation of BIRC5 to allow proper chromosome segregation^32^, and that targeted inhibition of PLK1 and BIRC5 inhibits the proliferation of bladder cancer cells^33^. BIRC5 is a FOXM1 target gene, and loss of FoxM1 induces cell death accompanied by decreased expression of the FOXM1 target genes BIRC5 and Bmi1^34^. FOXM1 overexpressing breast cancer cells displayed an anti-apoptotic phenotype due to up-regulated expression of XIAP and BIRC5 anti-apoptotic genes. Conversely, FOXM1 knockdown decreases XIAP and BIRC5 expression, as well as inhibits the binding of FOXM1 to the XIAP and BIRC5 promoter regions^35^.

Herein, we found that BIRC5 expression was elevated in the vast majority of tumors, with high expression levels positively correlating with shorter prognosis and level of immune cell infiltration in tumors. The study did not specifically address the molecular mechanisms associated with the marker genes, nor did it validate these targets using immunomodulatory drugs. However, the strength of this study is that it promotes the development of future immunotherapy research. In conclusion, this study shows that BIRC5 can be as a biomarker for use in the diagnosis and prognosis of cancers. The upregulation of BIRC5 in tumors was significantly and positively correlated with the level of activated tumor immune cells. This suggests an immunomodulatory role for BIRC5 in tumor immunity. We found that BIRC5 expression levels correlated with methyltransferase expression levels in different types of tumors, and the association between immune checkpoints and BIRC5 in cancer shows its potential as a therapeutic target. However, these discoveries should be validated by extension to large-scale genomic and functional studies.

## Materials and methods

### Process public sequencing data

Sequencing data for 33 tumors were downloaded from the TCGA (https://portal.gdc.cancer.gov/) database for 11,057 samples (10,327 tumor samples and 730 matched paraneoplastic samples). We searched the ONCOMINE (https://www.oncomine.org/) database for differences in tumor versus normal tissue expression of BIRC5 in common diseases, covering a total of 464 datasets from 20 tumor types (not subdivided into tumor subtypes). Given that there were too few paracancerous samples in some tumors, and that the error of the results may be too large when analyzing these tumors, we introduced a total of 5964 samples of healthy tissues and organs from the GTEx (https://www.gtexportal.org/home/) database. However, there were still some tumors with missing matches or too few paraneoplastic samples, and these tumors were excluded from the calculation of the difference in expression of target genes in tumor and paraneoplastic tissue, such as DLBC, MESO, PCPG, SARC, THYM, and UVM. For the remaining tumor samples, we performed a Wilcoxon test after normalization to analyze whether there is a difference in expression of BIRC5 between these tumors and healthy tissues. *P* < 0.05 indicates a statistically significant difference in expression.

### Correlation between BIRC5 expression and prognosis of tumor patients

We downloaded the survival times information of 10,327 tumor samples from 33 tumors in the TCGA (including Overall survival, Disease-specific survival, Disease-free interval, and Progression-free interval). Collated and divided each tumor sample into two high and low expression groups according to the median BIRC5 expression value, analyzed the prognostic value of BIRC5 in each tumor using the Cox test, and plotted the BIRC5 risk ratio forest plot. Second, we calculated the prognostic value of BIRC5 using the Kaplan–Meier survival estimate method, with indicators of significant differences determined by a log-rank test. Comprehensive analysis of the results obtained by these two methods, only when the overall survival test *P* < 0.05 and the four survival states in the Kaplan–Meier chart meet the difference simultaneously, we believe that the expression of BIRC5 significantly affects the prognosis of the tumor.

### Relationship between BIRC5 expression and tumor stage, TMB and MSI

We downloaded the pathological Stage information of all the tumor tissue samples and divided them into 3–4 groups according to the staging, leaving 8099 samples after removing samples with incomplete information. The limma package^36^ was used to calculate the expression of BIRC5 in each group and ggpubr package (https://CRAN.R-project.org/package=ggpubr) was used to plot the box line plot of the relationship between genes and tumor stage. We downloaded mutation data from a total of 10,114 samples of these 33 tumors in TCGA and calculated the mutation score for each sample to obtain tumor mutation load information for each tumor. Finally, we used the Spearman correlation test to analyze the correlation between BIRC5 expression and TMB and used the fmsb package (https://CRAN.R-project.org/package=fmsb) to create a correlation radar plot. We also downloaded and analyzed the MSI scores of 10,415 tumor samples, combined with the BIRC5 transcriptome data, and used the same method as above to plot the MSI correlation radar map between BIRC5 and tumors.

### Correlation analysis between tumor microenvironment and BIRC5 expression levels

Immune cells and stromal cells are the two main types of non-tumor components in the tumor microenvironment. They have been proposed to be have diagnostic and prognostic value in cancers^37,38^. ESTIMATE (https://bioinformatics.mdanderson.org/public-software/estimate/)^39^ is a tool for predicting stromal and immune cell infiltration abundance and tumor purity in tumor tissues using gene expression data. Based on the expression profile matrix files of 11,057 samples from 33 tumors, we estimated the tumor purity according to the proportion of stromal and immune cells in each tumor sample by using estimate and limma packages sequentially after removing the normal samples. The stromal score, immune score, and estimate score were used to calculate tumor purity. We then combined the BIRC5 expression data and the tumor microenvironment score data, calculated their correlations using the spearman correlation test, and plotted the correlations using the ggplot2 (https://CRAN.R-project.org/package=ggplot2), ggpubr, and ggExtra (https://CRAN.R-project.org/package=ggExtra) package to map the correlation distribution.

### Correlation analysis of immune cell infiltration and BIRC5 expression

CIBERSORT (https://cibersort.stanford.edu/)^40^ is a computational tool based on RNAseq data and used to estimate the relative percentage of infiltration of 22 immune cells (B cells naïve, B cells memory, plasma cells, T cells CD8, T cells CD4 naïve, T cells CD4 memory resting, T cells CD4 memory activated, T cells follicular helper, T cells regulatory (Tregs), T cells gamma delta, NK cells resting, NK cells activated, Monocytes, Macrophages M0, Macrophages M1, Macrophages M2, Dendritic cells resting, Dendritic cells activated, Mast cells resting, Mast cells activated, Eosinophils and Neutrophils) in a tumor^41^. Similarly, based on the full tumor expression matrix file, after initial processing and correction with the limma package, we used the CIBERSORT algorithm in R software to calculate the 22 immune cell infiltration scores for each sample in the tumor. After selecting the tumor samples, we used Spearman's correlation test to analyze the correlation between the individual infiltration levels of the 22 immune cells in the 33 tumors and BIRC5 expression.

### Protein–protein interaction networks and gene enrichment analysis

Having obtained results on the correlation of BIRC5 with the tumor microenvironment and immune cell infiltration, we sought to understand the molecular mechanisms underlying their intrinsic association and their collective impact on the tumor. We constructed a network of 31 co-expressed genes, including BIRC5 in the STRING V11.0 (http://string-db.org)^42^ database. We analyzed their roles and mechanisms in tumors in the Metascape (https://metascape.org/) database. Metascape is a reliable online analysis tool that provides a comprehensive annotated list of genes and resources for real-time analysis and is updated monthly^43^. Based on the annotated lists of KEGG Pathway (https://www.kegg.jp/kegg/kegg1.html)^44^, GO Biological Processes, Reactome Gene Sets, Canonical Pathways, and CORUM, we enriched these genes into clusters. Terms with a *P* value < 0.01, a minimum count of 3, and an enrichment factor > 1.5 (the enrichment factor is the ratio between the observed counts and the counts expected by chance) are collected and grouped into clusters based on their membership similarities. *P* values are calculated based on the accumulative hypergeometric distribution.

### Correlation of BIRC5 with known essential marker genes

Having explored the relationship between BIRC5 and the tumor microenvironment and immune cell infiltration, we wanted to investigate the mechanism by which they are linked or associated with immune checkpoint genes. We synthesized 47 immune checkpoint genes from the literature and analyzed the correlation between BIRC5 expression and the expression of these immune checkpoint genes in 33 tumors sequentially using the limma package and spearman's test. The reshape2 package (http://www.jstatsoft.org/v21/i12/) was used to create a correlation heat map. DNA methylation alters chromatin structure, DNA conformation, DNA stability, and the way DNA interacts with proteins without altering the DNA sequence alterations. Accordingly, we analyzed the correlation between the expression of BIRC5 and four methyltransferases (DNMT1, DNMT2, DNMT3A, and DNMT3B)^45^. The analyses were performed in much the same way as described above. To determine the most significant Gene Ontology function of BIRC5 in each tumor, the tumors were grouped into high and low expression groups based on the expression level of BIRC5. The expression level of BIRC5 was then analyzed by limma, org.Hs.eg.db, clusterProfiler (http://bioconductor.org/packages/release/bioc/html/clusterProfiler.html), and enrichplot package based on c5. all.v7.1.symbols background file to perform Gene Set Enrichment Analysis on the tumor expression matrix file and the top 5 most significant GOs were selected to plot enrichment curves. *P* < 0.05 was considered a significant difference criterion.

### Statistical analysis

The R version 3.6.1 software (https://www.r-project.org/) and its ancillary packages were used for data analysis. Limma package and Student's t-test were used to analyze BIRC5 expression, and *P* < 0.05 was considered statistically significant. Kaplan–Meier curves were used for survival analysis using the log-rank test, and *P* < 0.05 was considered statistically significant survival difference. Spearman or partial Spearman method was used to analyzing correlations between genes and correlations between genes and immune cells. *P* < 0.05 was considered statistically significant. All figures in this study were performed using R version 3.6.1 (2019-07-05)^46^.

## Supplementary information

Supplementary Figure S1.

Supplementary Figure S2.

Supplementary Figure S3.

Supplementary Figure Legends.

## Data Availability

These data are drawn from the following resources in the public domain. TCGA (https://cancergenome.nih.gov/), GTEx (https://www.gtexportal.org/home/), ONCOMINE (https://www.oncomine.org/), STRING (https://string-db.org/), Metascape (https://metascape.org/). All data in this study were permitted for use.
