# Supplementary figures and images for: BIRC5 is a prognostic biomarker associated with tumor immune cell infiltration
Source: Sci Rep. 2021 Jan 11;11:390. doi: 10.1038/s41598-020-79736-7 (PMC7801710; doi:10.1038/s41598-020-79736-7)

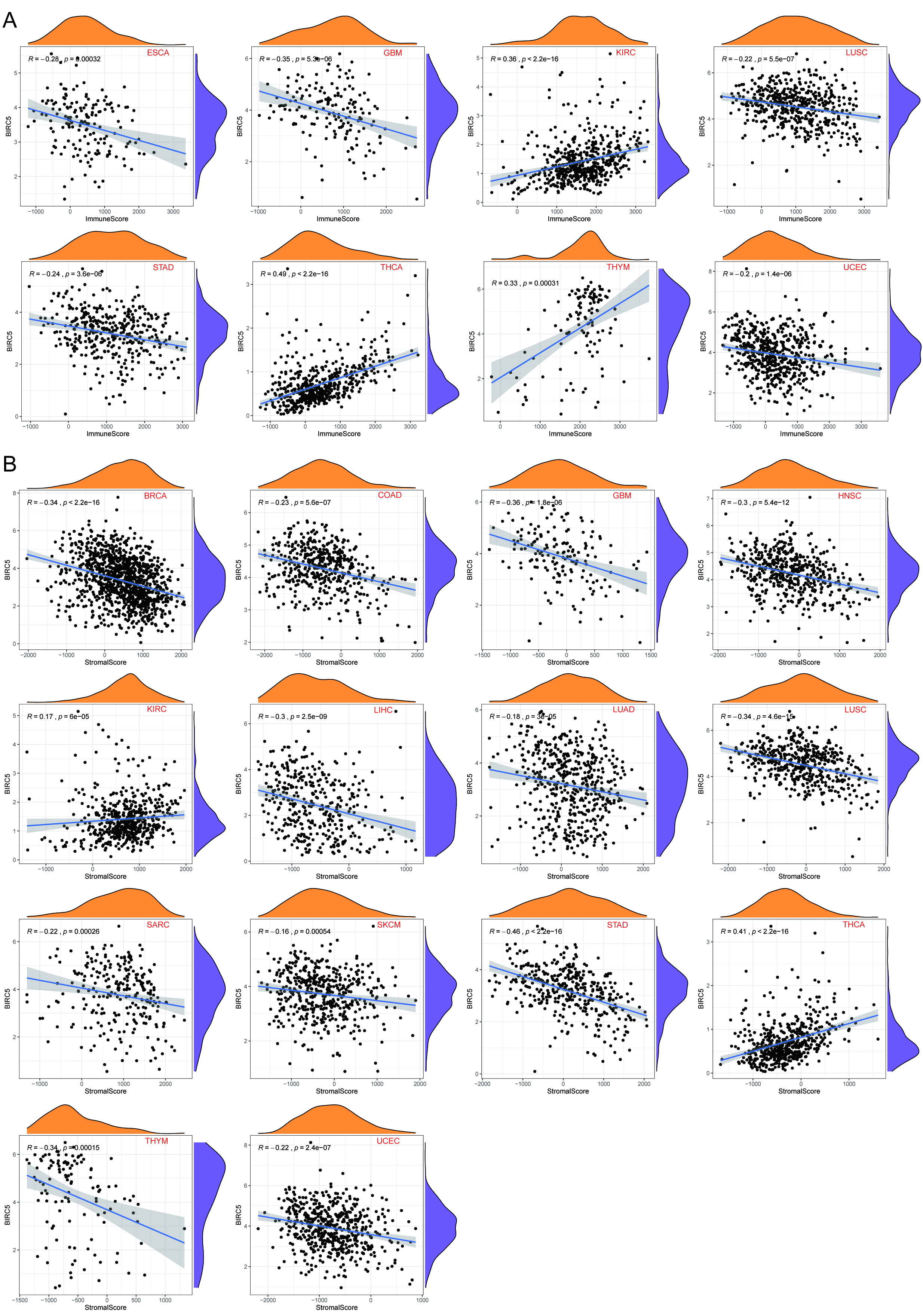

Supplement: Supplementary file 1 — Supplementary Figure S1. [file 41598_2020_79736_MOESM1_ESM.tif]

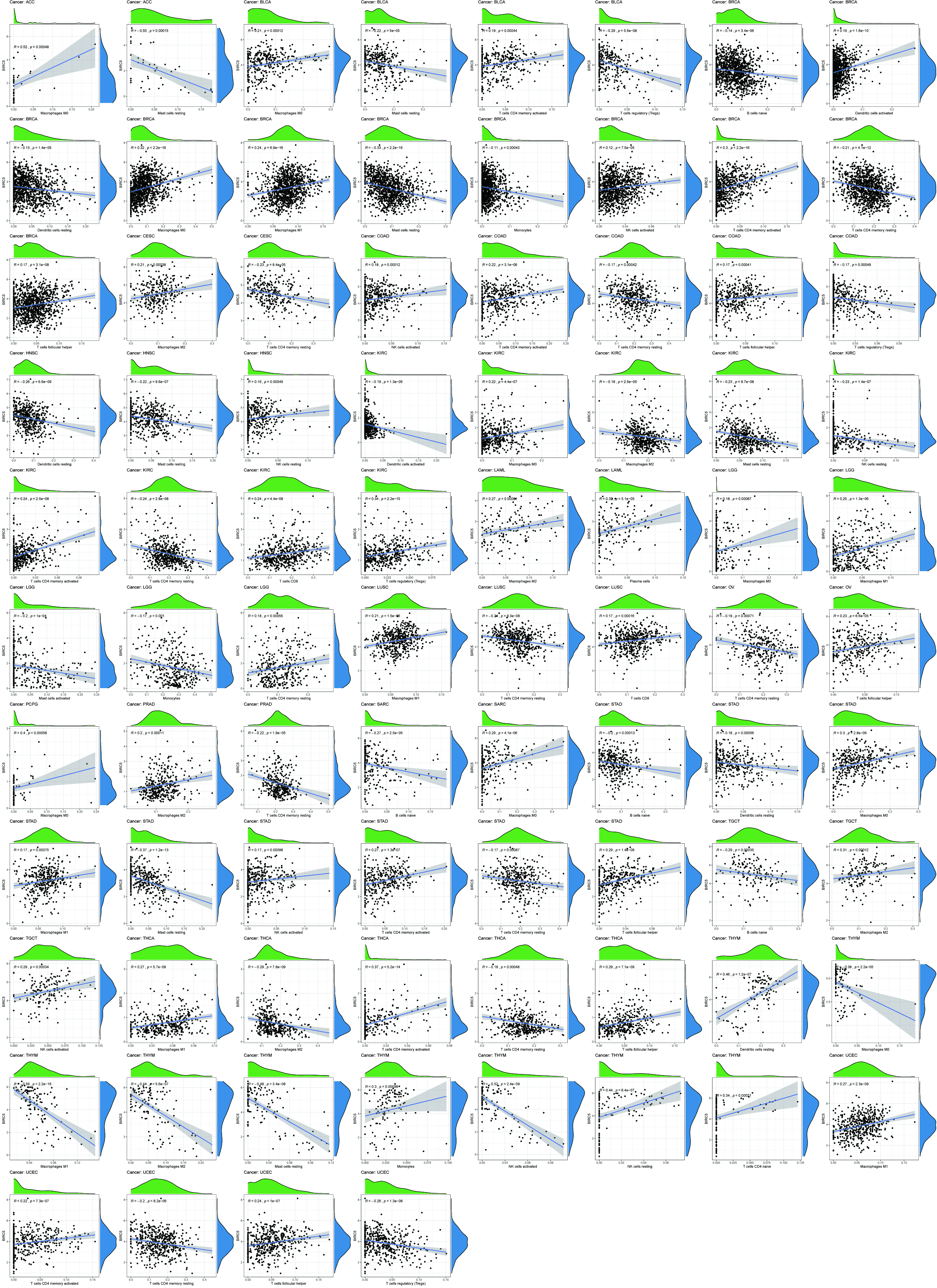

Supplement: Supplementary file 2 — Supplementary Figure S2. [file 41598_2020_79736_MOESM2_ESM.tif]

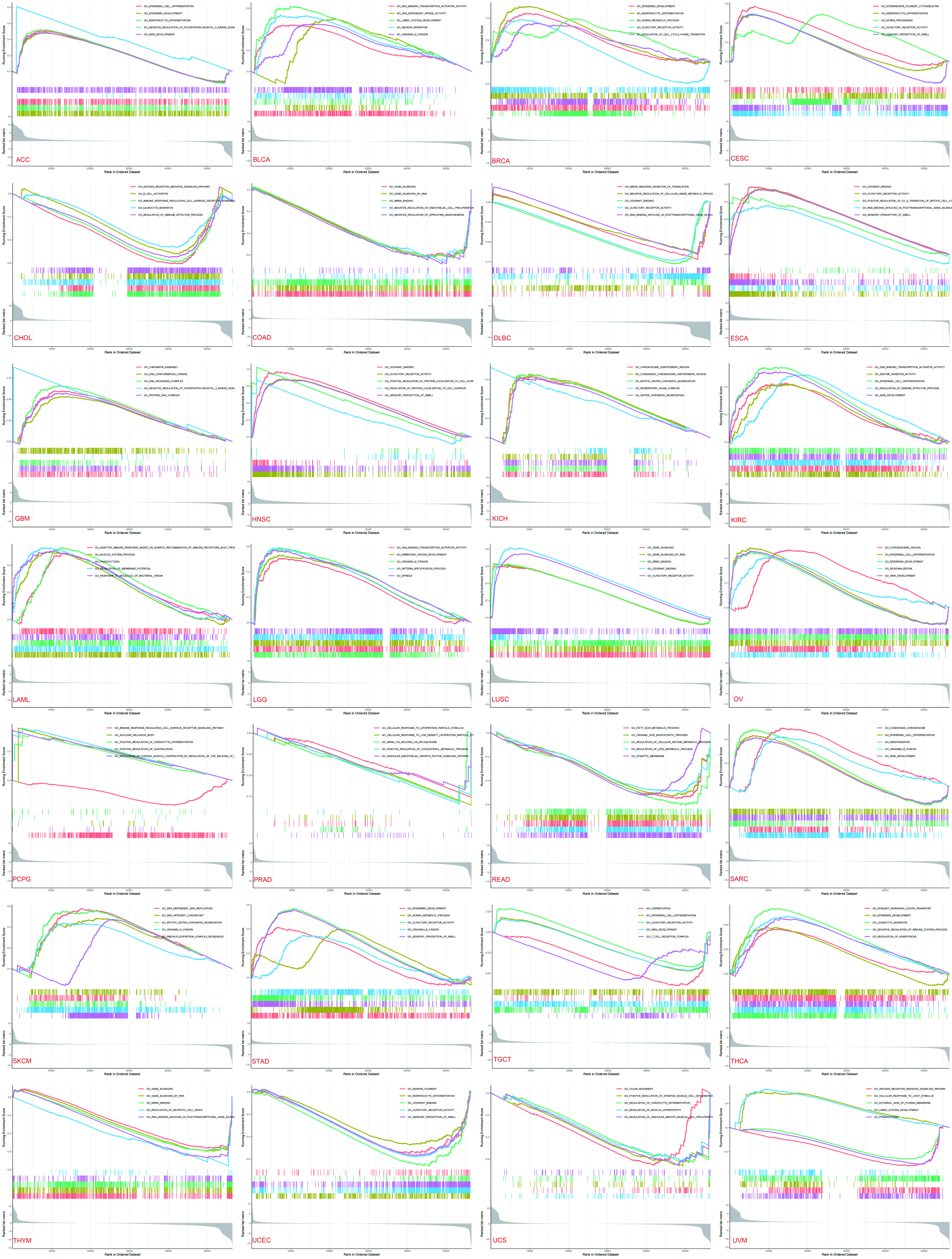

Supplement: Supplementary file 3 — Supplementary Figure S3. [file 41598_2020_79736_MOESM3_ESM.tif]
