# Supplementary material for: BIRC5 is a prognostic biomarker associated with tumor immune cell infiltration
Source: Sci Rep. 2021 Jan 11;11:390. doi: 10.1038/s41598-020-79736-7 (PMC7801710; doi:10.1038/s41598-020-79736-7)
Supplement: Supplementary file 4 — Supplementary Figure Legends. [file 41598_2020_79736_MOESM4_ESM.docx]

**Supplementary Figure Legends**

**Fig. S1.** The relationship between BIRC expression level and tumor microenvironment (TME) of different types of cancers. **(A)** the relationship between BIRC5 expression level and Immune Score. **(B)** the relationship between BIRC5 expression level and Stromal Score. The figure was performed using R version 3.6.1 (2019-07-05)^46^.

**Fig. S2.** The relationship between the expression level of BIRC5 and the infiltration level of different kinds of immune cells in patients with other cancers. The figure was performed using R version 3.6.1 (2019-07-05)^46^.

**Fig. S3.** GSEA analysis based on BIRC5 in other cancers. The figure was performed using R version 3.6.1 (2019-07-05)^46^.
